# Minimising young children’s anxiety through schools (MY-CATS): protocol for a cluster randomised controlled trial to evaluate the effectiveness and cost-effectiveness of an online parent-led intervention compared with usual school practice for young children identified as at risk for anxiety disorders

**DOI:** 10.1186/s13063-022-06010-8

**Published:** 2022-02-16

**Authors:** Tessa Reardon, Helen Dodd, Claire Hill, Bec Jasper, Peter J. Lawrence, Fran Morgan, Ronald M. Rapee, Obioha C. Ukoumunne, Mara Violato, Emily Davey, Gemma Halliday, Benjamin Jones, Lindsey Martineau, Amy McCall, Natascha Niekamp, Anna Placzek, Ruth Potts, Tamatha Weisser, Cathy Creswell

**Affiliations:** 1 Departments of Experimental Psychology and Psychiatry, University of Oxford, Oxford, UK; 2 College of Medicine and Health, University of Exeter, Exeter, UK; 3 School of Psychology & Clinical Language Sciences, University of Reading, Reading, UK; 4 Parents and Carers Together, Suffolk, UK; 5 Centre for Innovation in Mental Health, School of Psychology, University of Southampton, Southampton, England; 6 Square Peg, East Sussex, UK; 7 Centre for Emotional Health, Department of Psychology, Macquarie University, Sydney, Australia; 8 NIHR ARC South West Peninsula (PenARC), University of Exeter, Exeter, UK; 9 Health Economics Research Centre, Nuffield Department of Population Health, University of Oxford, Oxford, UK

**Keywords:** Anxiety, Children, Prevention, Early intervention, Screening, Schools, At risk, Online intervention, Parent-led intervention, Cost-effectiveness

## Abstract

**Background:**

Identifying and supporting young children who are at risk of developing anxiety disorders would benefit children, families, and wider society. Elevated anxiety symptoms, inhibited temperament, and high parental anxiety are established risk factors for later anxiety disorders, but it remains unclear who is most likely to benefit from prevention and early intervention programmes. Delivering an online intervention through schools to parents of young children who have one or more of these risks could maximise reach. The primary aim of this trial is to evaluate the effectiveness and cost-effectiveness of delivering an online parent-led intervention, compared with usual school provision only, for children (aged 4–7) identified as at risk for anxiety disorders on the basis of at least one risk factor. We also aim to identify the characteristics of children who do and do not benefit from intervention and mechanisms of change from the intervention.

**Methods:**

The design will be a parallel group, superiority cluster randomised controlled trial, with schools (clusters) randomised to intervention or usual school practice arms in a 1:1 ratio stratified according to level of deprivation within the school. The study will recruit and randomise at least 60 primary/infant schools in England, and on the basis of recruiting 60 schools, we will recruit 1080 trial participants (540 per arm). Parents of all children (aged 4–7) in sampled Reception, Year 1, and Year 2 classes will be invited to complete screening questionnaires. Children who screen positive on the basis of anxiety symptoms, and/or behavioural inhibition, and/or parent anxiety symptoms will be eligible for the trial. Parents/carers of children in schools allocated to the intervention arm will be offered a brief online intervention; schools in both arms will continue to provide any usual support for children and parents throughout the trial. Assessments will be completed at screening, baseline (before randomisation), 6 weeks, 12 weeks, and 12 months post-randomisation. The primary outcome will be the absence/presence of an anxiety disorder diagnosis at 12 months.

**Discussion:**

The trial will determine if delivering an online intervention for parents of young children at risk of anxiety disorders identified through screening in schools is effective and cost-effective.

**Trial registration:**

ISRCTN 82398107. Prospectively registered on Jan. 14, 2021.

**Supplementary Information:**

The online version contains supplementary material available at 10.1186/s13063-022-06010-8.

## Background

Anxiety disorders are the most common mental health disorders experienced across the lifespan [1]. Half of all lifetime cases begin by age 11 [1], and the estimated prevalence worldwide is 6.5% [2]. Children with anxiety disorders are more likely than their peers to experience ongoing anxiety problems and other serious mental health disorders and have reduced educational and employment opportunities [3]. Due to the high prevalence and ongoing impact on children and their families, anxiety disorders have higher societal costs than any other mental health disorder [4].

Only a minority of children with mental health problems access treatment [5], with extensive barriers to identification and treatment access widely reported [6, 7]. Intervening before anxiety difficulties become ingrained would minimise the consequences for children, their families, and society. One option for prevention and early intervention is to take a universal approach that targets a whole population. However, there is evidence that these interventions may not benefit children with high anxiety symptoms, who need them the most [8]. An alternative approach is to target interventions at children who are *most* likely to develop anxiety disorders; emerging evidence supports the value of this approach [9].

### Whom should prevention programmes target?

In addition to elevated anxiety symptoms, the two most robust predictors of the development of anxiety disorders in children are inhibited temperament (the tendency to withdraw, avoid, or respond fearfully to new situations) which increases the risk of later anxiety disorders threefold [10], and having a parent with an anxiety disorder, which raises the risk almost twofold [11]. Furthermore, these risk factors may affect child anxiety in an additive way [12]. There is evidence that prevention can be effective when children are identified on the basis of elevated anxiety symptoms [13], behavioural inhibition [14], and parental anxiety disorders [15]. To date, programmes have typically identified children on the basis of a single risk factor [9] and so have been unable to establish whether the presence of one particular risk factor or combination of risk factors can determine who is most likely to benefit from targeted prevention and early intervention.

### What should prevention programmes target?

*Cool Little Kids* [14] is the most extensively evaluated anxiety prevention programme for young children and focuses on strategies that parents can implement with their child to encourage independence, reduce parent overprotection, and manage child avoidant coping. However, change in intervention targets are not consistently reported [16, 17], and the mechanisms of change from the intervention remain unclear. Indeed, other key potential mechanisms of change from parent-focused programmes for young children include parenting efficacy [18] and child targets, including coping efficacy [19] and tolerance of uncertainty [20]. Identifying mediators of intervention outcomes would allow us to optimise the content and focus of future interventions.

### How should prevention programmes be delivered?

Systematically screening young children and offering a brief intervention for those identified as at risk could maximise the reach of prevention. To date, one study has incorporated universal screening (for behavioural inhibition) into a population-level face-to-face prevention programme finding evidence of significant reductions in internalizing symptoms [16] and preliminary evidence of cost-effectiveness [21]. However, face-to-face intervention delivery is a barrier to widespread implementation. Online delivery has potential to improve the reach of mental health interventions, as long as participant engagement is maximised through the development and delivery process [22]. Together with parents, children, and clinicians, we have developed an online version of an effective and cost-effective intervention for child (7–12 years) anxiety disorders called OSI (Online Support and Intervention for child anxiety) [23–25]. OSI was originally developed for use in NHS (National Health Service) clinics [25], and we have since adapted the content for children with anxiety problems identified through screening in primary schools [26]. In consultation with parents, we have now further adapted the content of OSI for parents/carers of children (aged 4–7) who are at risk of developing anxiety disorders to motivate and help parents/carers to develop and use skills to prevent child anxiety problems emerging.

### Aims and objectives

Our primary aim is to evaluate the effectiveness and cost-effectiveness of the provision of an online parent-led intervention (OSI) compared with usual school practice for children (aged 4–7) identified as at risk for anxiety disorders on the basis of screening positive for one or more risks (anxiety symptoms, and/or behavioural inhibition, and/or parental anxiety). We want to determine whether providing OSI to at-risk children identified through systematic screening in schools brings benefits over and above usual school practice. We also set out to identify the characteristics of children who do and do not benefit from the intervention and the mechanisms of change from the intervention.

Our primary objective is to compare diagnostic outcomes 12 months post-randomisation for children who screen positive on one or more risk factors in schools allocated to intervention versus usual school practice, and we hypothesise that fewer children will have an anxiety disorder in the intervention arm compared to the usual school practice arm at 12 months.

Secondary objectives are:

- To compare anxiety symptoms, related interference, externalising symptoms, and additional intervention targets 12 weeks and 12 months post-randomisation for children who screen positive on one or more risk factors in schools allocated to intervention compared to usual school practice.
- To identify moderators (including number of risks) and mediators of the primary outcome.
- To evaluate experiences of systematic screening and the parent-led online intervention.
- To estimate the cost-effectiveness of the intervention compared to usual school practice at 12 months post-randomisation.

This protocol follows the Standard Protocol Items: Recommendations for Interventional Trials (SPIRIT) [27] (see Additional file 1 for SPIRIT checklist).

## Methods

### Study design

The study will use a parallel group, superiority cluster randomised controlled trial design, with schools (clusters) randomised to the intervention or usual school practice arm in a 1:1 ratio stratified according to level of deprivation within the school. Cluster randomisation will prevent potential contamination between parents in the same school. Parents/carers of all children (aged 4–7) in sampled Reception, Year 1, and Year 2 classes will be invited to take part in screening. Children who screen positive on the basis of child anxiety symptoms, and/or behavioural inhibition, and/or parent anxiety symptoms will be eligible for the trial. As the intervention is parent-led, a maximum of one child per family/household will be eligible for the trial, and where more than one child in a family/household screens positive on at least one risk, one child will be invited to take part on the basis of screening scores (see below). Parents/carers of children in schools allocated to the intervention arm will be offered a supported, parent-led online intervention (OSI: Online Support and Intervention for child anxiety), and families in schools allocated to usual school practice will be offered written versions of the online intervention at the end of the trial. Schools in both arms will continue to provide any usual support throughout. Assessment points will be screening, baseline (prior to randomisation), 6 weeks (potential mediators only), 12 weeks, and 12 months post-randomisation. Qualitative interviews will be used to explore parents, children, and school staff’s experiences of screening and the intervention. Schools and participants will be recruited in up to four recruitment cohorts, with recruitment and data collection expected to take place from February 2021 to at least January 2023.

### Setting

The study setting is mainstream primary/infant schools in England with at least two Reception, Year 1, and Year 2 classes (children aged 4–7). Where schools have more than two classes in one or more target year groups, two classes per target year group will be randomly sampled.

### Participants and eligibility

#### Inclusion criteria for screening

- Child (aged 4–7) is in a participating class (Reception, Year 1, Year 2).
- Child’s parent/carer provides written consent and completes screening questionnaires for the child.

#### Inclusion criteria for the trial

- Child screens positive on the basis of at least one of the following screening questionnaires: child anxiety symptoms (score ≥ 34 on the Preschool Anxiety Scale; PAS), behavioural inhibition (score ≥ 30 on the Approach subscale of the Short Temperament Scale for Children; STSC-A), parent/carer anxiety symptoms (score ≥ 8 on the Generalised Anxiety Disorder-7 Scale).
- Child’s parent/carer provides written consent.
- A maximum of one child per family/household will be eligible to participate in the trial. Where more than one child in a family/household screens positive on the basis of at least one screening questionnaire, the child with the highest score on the PAS will be eligible. If two or more children in the family/household have equal PAS scores, the child with the highest STSC-A score will be eligible.

#### Exclusion criteria for the trial

- Parent/carer does not have sufficient use of English to provide consent, complete measures, and/or take part in the intervention.
- Parent/carer does not have frequent access to the internet, either at home or elsewhere.

#### Inclusion criteria for qualitative interviews

- Parents: parent/carer takes part in screening and provides written consent, including consent for audio recording the interview.
- Children: child of parent/carer who takes part in the intervention provides written assent, and their parent/carer provides written consent, including consent/assent for audio recording the interview.
- School staff: member of staff at a participating school and they provide written consent, including consent for audio recording the interview.

### Recruitment

We will recruit schools and participants in recruitment cohorts. We anticipate each recruitment cohort will start screening/baseline in a new school term and expect to recruit up to four cohorts of schools/participants.

#### School recruitment

We aim to recruit a sample of mainstream primary/infant schools in England that includes variation in relation to geographic location, size of school and demographic profile (percentage of pupils eligible for free school meals, percentage of pupils on special educational needs support, percentage of pupils with English as an additional language). We will monitor the characteristics of recruited schools and target particular schools as needed to try to maximise the representativeness of primary schools in England.

A range of strategies will be used to recruit schools, drawing on procedures we have used previously to successfully recruit a large number of primary schools [28, 29]. Publicly available information will be used to identify potentially eligible schools from a range of geographic areas, and we will contact school staff via email and follow-up telephone/online calls. We will also disseminate information about the study via our existing networks, presentations at events and conferences, and social media.

We will obtain written agreement from school headteachers and participating schools will nominate a study lead to liaise with the research team and help facilitate participant recruitment. Schools will be asked to provide information on eligible classes (class name, number of registered children). Where a school has more than two eligible classes in a target year group, two classes per year target group will be randomly selected to participate.

#### Participant recruitment

School staff will distribute electronic versions of study information to parents/carers of all children in participating classes. Together with each school, we will develop strategies to help promote participation in screening (e.g. distributing video adverts, advertising the study on the school website/newsletter, distributing paper versions of study information to supplement electronic information, sending reminder emails, researchers running online information sessions, recruiting parent champions in the school to help promote the study, distributing stickers with the study logo, displaying study adverts in the school). Parents/carers will provide written consent via an online survey prior to completing screening questionnaires.

We will provide parents/carers with written feedback informing them whether screening questionnaire responses indicate their child is eligible for the trial or not. Where a child is eligible for the trial, we will also arrange a follow-up telephone call with the parent/carer to discuss the trial in more detail, answer any questions, and invite them to take part. With parental consent, we will share a list of children eligible for the trial with school staff.

Parents/carers who verbally agree to the trial will be asked to provide written consent online and complete baseline questionnaires.

### Data collection procedures

**Fig. 1:**
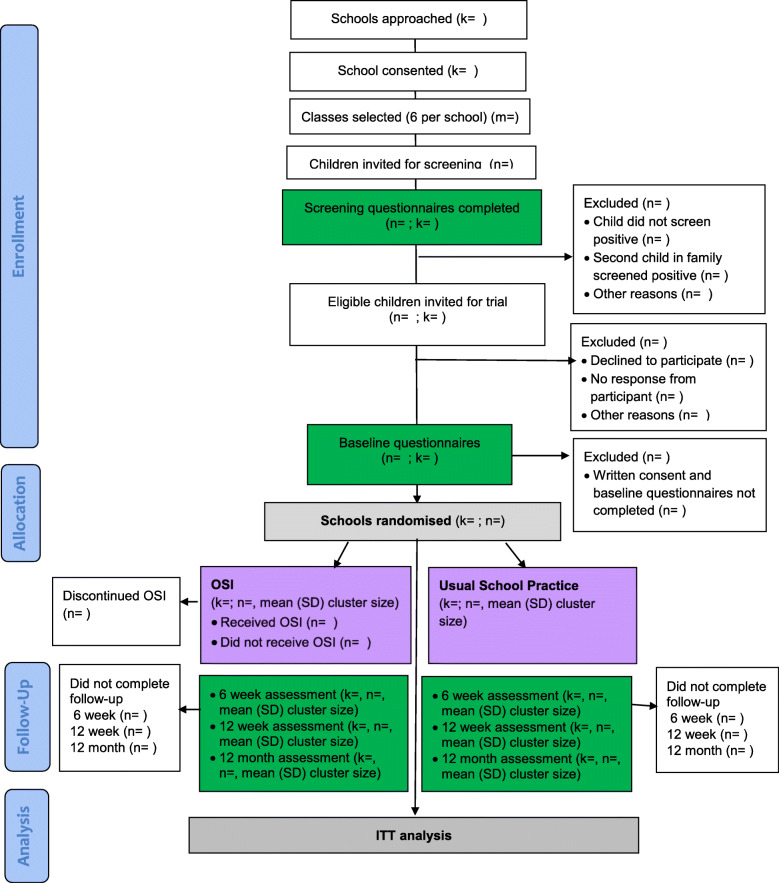
Consolidated Standards for Reporting Trials (CONSORT) diagram

**Fig. 2:**
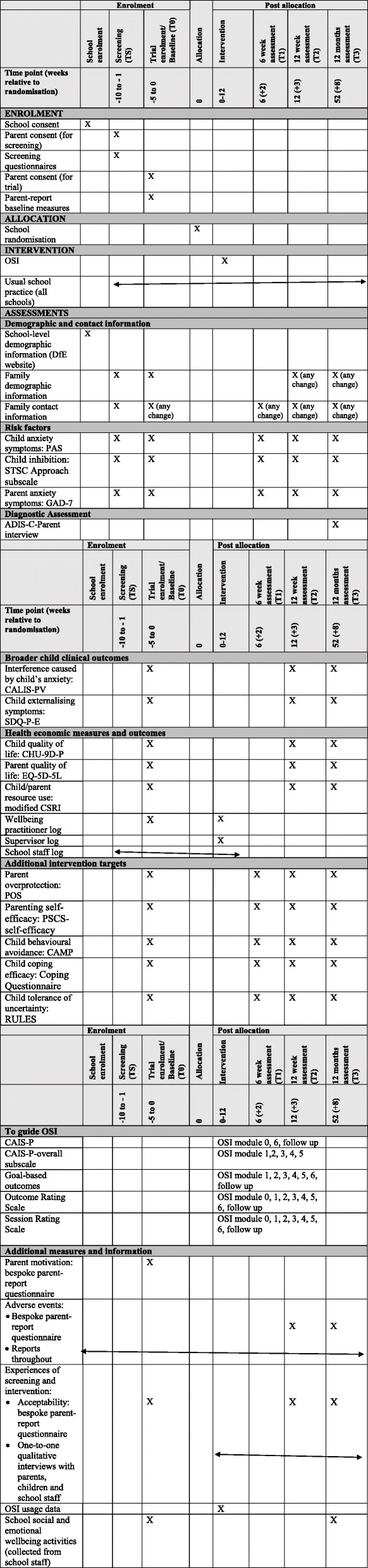
Schedule of enrolment, intervention and assessment. *Note.* TS = Screening assessment; T0 = Baseline assessment; T1 = 6-week post-randomisation assessment; T2 = 12-week post-randomisation assessment; T3 = 12-month post-randomisation assessment; OSI = Online Support and Intervention for child anxiety; DfE = Department for Education; PAS = Preschool Anxiety Scale; STSC Approach subscale = Short Temperament Scale for Children-Approach subscale; GAD-7 = Generalised Anxiety Disorder Scale-7; ADIS-C-Parent interview = Anxiety Disorder Interview Schedule for Children-Parent interview; CALIS-PV = Child Anxiety Life Interference Scale -Preschool version; SDQ-P-E=Strengths and Difficulties Questionnaire-Parent report-Externalising scale; CHU-9D=P = Child Health Utility-9D-Parent-report; EQ-5D-L = EuroQuality of life-5 level instrument; CSRI = Client Service Receipt Inventory; POS = Parental Overprotection Scale; PSCS-self-efficacy = Parenting Sense of Competence Scale-self-efficacy subscale; CAMP = Child Avoidance Measure-Parent report; RULES = Responses to Uncertainty and Low Environmental Structure; CAIS-P = Child Anxiety Impact Scale-Parent report

Figures 1 and 2 provide an overview of study procedures and assessments.

Parents/carers will complete questionnaire measures at screening and baseline (prior to randomisation) and follow-up assessments (6 weeks, 12 weeks, and 12 months post-randomisation) via online surveys. At the 12-month assessment, parents/carers will also take part in a diagnostic assessment for their child. Diagnostic interviews will be administered via telephone or online video call, and with parental consent will be audio-recorded.

At baseline and 12 months, school staff will be asked to provide information on activities related to social, emotional, and mental health and wellbeing offered in their schools in target year groups.

We will also collect information on time spent on activities related to screening and delivering the intervention. School staff will be asked to complete a log to record time spent on activities related to screening, and study therapists (Children’s Wellbeing Practitioners) and supervisors will complete logs to record time spent on activities related to delivering the intervention.

We will conduct one-to-one interviews with parents/carers, children, and school staff to explore experiences of participating in screening and the intervention. We anticipate that we will interview up to approximately 30 parents/carers, including parents/carers of children who are not eligible for the trial, parents/carers in the intervention arm, and parents/carers in the usual school practice arm; up to 10 children in the intervention arm; and up to 10 members of staff from participating schools across both arms. Written consent, including explicit consent for audio recording will be obtained prior to parent/school staff qualitative interviews, and written parental consent and child assent will be required for interviews with children.

Schools and families will be offered payments as a reimbursement for time spent on study activities. Schools will be offered £3 for each set of completed baseline questionnaires, families will be offered a £10 gift voucher for completing each follow-up assessment, and £10 will be offered to qualitative interview participants. To help maximise retention to the assessment schedule, we will also send all schools and families regular study updates and newsletters, and SMS/email/telephone reminders to complete online measures.

Standard operating procedures for managing significant distress/risk/safeguarding concerns will be followed throughout, and any potential adverse events will be recorded and managed in accordance with the study adverse events protocol.

### Sample size

Our target sample size is 1080 ‘at risk’ children from 60 schools; 30 schools (clusters) will provide a total of 540 children per arm. Six classes per school (two classes per target year group) will be invited to take part in screening (estimated 30 children per class, 10,800 children in total). Where schools have more than two classes in one or more target year group, classes will be chosen randomly, using computer-generated random numbers. This sample is large enough to detect a reduction in the presence of anxiety disorders at 12 months (primary outcome) from 50% (usual school practice arm) to 35% (intervention arm) with 90% power at the 5% (2-sided) level. This difference would be meaningful to detect and is in line with outcomes reported in previous positive child anxiety prevention trials (e.g. [17]).

The sample size calculation assumes (i) 50% of invited children participate in screening (5400 of 10,800), (ii) 20% of those who participate in screening, screen positive, and (iii) 80% of those that screen positive complete the 12-month follow-up. It also allows for clustering within schools, assuming an intra-cluster (intra-school) correlation coefficient of 0.05. Fifty percent participation is a conservative estimate based on a recent UK school-based study in which 72% of parents responded to similar class-wide screening questionnaires [30]. Previous studies indicate 10–15% will score above the cut-off on each single screen [16, 31, 32]; we estimate 20% will screen positive on at least one risk factor [12]. The median intra-cluster (intra-school) correlation coefficient in a recent systematic review of school-based cluster randomised trials was 0.028 [33]. Our assumed value of 0.05, therefore, leans on the side of caution.

As we will recruit schools and participants in recruitment cohorts, we will have the opportunity to monitor recruitment rates and review the sample size assumptions after each cohort. The sample size calculation is based on a minimum number of participating schools, so if there are insufficient participants recruited in initial cohorts, it will be possible to increase the total number of schools as required. If we need to increase the number of schools, the total number of trial participants we will require to detect the same effect size may also change.

### Randomisation and concealment

Schools will be randomised to the intervention (OSI) or usual school practice arm in a 1:1 ratio stratified according to level of deprivation in the school (above/below national average of 15.8% for percentage of pupils eligible for free school meals) using a blocked randomisation approach with block sizes of two and four. In order to minimise any imbalance between trial arms in terms of number of pupils, the schools in each recruitment cohort will be ordered by the number of recruited pupils prior to allocation. An independent statistician will conduct randomisation via a computer-generated algorithm. Schools will be randomised en bloc, after screening, participant enrolment, and baseline assessments have been completed in a cohort of schools. The independent statistician will pass the allocation to the Trial Manager, and the research team will inform schools and parents/carers which arm they are allocated to.

It is not possible for participants or study therapists to be blind, but all assessors administering diagnostic assessments and their supervisors will be blind to participant arm. We will explain assessor blinding to participants and ask them not to reveal their trial arm during the assessment. We will also ask the assessor after each interview whether they became unblinded. Study statisticians will remain blind to school/participant trial arm at least until completion of the primary analysis of the primary outcome. Health economists will need to be unblinded in order to conduct economic analyses.

### Interventions

#### OSI: Online Support and Intervention for child anxiety

Parents/carers of children in schools allocated to the intervention arm will be offered a brief, parent-led online intervention (OSI: Online Support and Intervention for child anxiety) [25]. OSI is an online version of an evidence-based parent-guided treatment for child anxiety disorders [23, 24], and we have adapted the content for parents/carers of children (aged 4–7) at risk of developing anxiety disorders. OSI includes seven online modules for parents, supported by weekly short telephone sessions (approximately 20 min) with a Children’s Wellbeing Practitioner (CWP), and a follow-up review telephone session 4 weeks later. Modules teach parents skills and strategies to help prevent and manage child anxiety problems, using principles of psychoeducation, promoting independence, fear testing, and problem solving. Modules include simple text, audio versions of text, videos, animations, interactive activities and inbuilt questionnaires, and CWPs can view responses and activities parents complete online via an accompanying practitioner website. Parents/carers will also be offered an accompanying optional game app for children that is designed to help motivate children to face their fears. For the purpose of analyses examining the effect of compliance with the intervention (detailed below), a ‘complier’ will be defined as a participant who completes a minimum of the first five online modules.

We expect four CWPs will support OSI delivery in this trial. CWPs (NHS, Band 5) are trained to deliver low-intensity psychological therapies for children and young people with anxiety problems, low mood, and behavioural problems. Trial CWPs will follow highly structured and standardised guidance on how to support parents to work through the online modules, practice skills and strategies, and problem solve, and will receive weekly supervision from a clinical psychologist with expertise in delivering interventions for child anxiety problems following initial training in the parent-led CBT approach generally and OSI specifically. Training will be conducted through reading, discussion, observation, and role-play. Adherence will be closely monitored throughout the trial by the supervising clinical psychologists, and with parental consent, telephone sessions will be audio-recorded for use in supervision.

#### Usual school practice

This trial aims to establish whether offering a brief, parent-led intervention to parents/carers of children at risk of developing anxiety disorders provides additional benefit beyond usual, current practice. Families in both arms will be free to continue to seek and access social, emotional, and mental health support and intervention for their child, and schools in both arms will continue to provide any usual social, emotional, and mental health support offered to children and families in their school. After the 12-month assessment, parents/carers in the usual school practice arm will be offered written versions of the content of the OSI website.

### Screening and outcome measures

Details of measures and timings are provided in Fig. 2.

#### Screening measures

- Child anxiety symptoms will be assessed using the Preschool Anxiety Scale (PAS) [32]. The PAS consists of 28 items and for each item parents select a response that best describes their child using a 5-point Likert scale. Items are scored 0–4 and responses summed to produce a total score (range 0–112). Children who score ≥ 34 will screen positive on the basis of published norms for 3- to 6.5-year-olds (https://www.scaswebsite.com). Some children will be aged 7 at screening (and 7–8 at follow-up), but we will use the PAS throughout to facilitate comparison across the sample and time points.
- Behavioural inhibition will be measured using the 7-item Approach subscale of the Short Temperament Scale for Children (STSC-A) [34, 35]. Parents rate each item on a 6-point scale (scored 1–6, including 4 reverse scored items) which are summed to provide a total score (range 7–42). In line with previous prevention trials (e.g. [16]), and consistent with laboratory assessments of behavioural inhibition [36], children will screen positive if they score ≥ 30.
- Parent anxiety symptoms will be assessed using the 7-item Generalised Anxiety Disorder Scale (GAD-7) [37]. The GAD-7 is the recommended screening tool for detecting anxiety disorders in adults [38]. Parents rate each item using a 4-point (0–3) response scale (total score range 0–21) with reference to the previous 2 weeks, and scores ≥ 8 [39] will be classified as screen positive.

#### Primary outcome

Our primary outcome is the absence/presence of an anxiety disorder at 12 months post-randomisation established on the basis of the Anxiety Disorder Interview Schedule for Children Parent interview (ADIS-P) [40]. The ADIS is the most widely used assessment to establish anxiety disorder status in child anxiety treatment trials [41], and the parent interview version can be administered by telephone to reliably establish anxiety disorder status in young children [42]. We are expecting an updated version of the ADIS-P interview schedule will be available for use in this trial, and if not we will consult with the authors to make minor amendments to the existing interview schedule to enable diagnoses of anxiety disorders and common comorbid disorders consistent with Diagnostic and Statistical Manual of Mental Disorder-5 (DSM-5). Assessors who are blind to school/participant trial arm will administer diagnostic interviews with parents/carers via telephone or video call. Assessors will be psychology graduates trained through discussion, observation, and role-play. Standard guidance will be followed to assign diagnoses and Clinical Severity Ratings (CSRs) based on the parent interview. Diagnoses and CSRs 4–8 will be assigned if a child meets the diagnostic criteria, and CSRs 0–3 will be used to assign sub-clinical diagnoses. For each assessor, diagnoses and CSRs will be reviewed by a consensus team led by an experienced diagnostician for at least the first 20 interviews. Inter-rater reliability will be assessed, and once assessors demonstrate high inter-rater reliability (kappa for presence/absence of diagnosis ≥ 0.85; intra-class correlation for CSRs ≥ 0.85), one in six interviews will be reviewed to prevent assessor rater drift.

### Secondary outcomes

#### Child clinical outcomes

The following secondary child clinical outcomes will be assessed at baseline, 12 weeks, and 12 months post-randomisation:

- Child anxiety symptoms measured using the PAS total score.
- Interference related to the child’s anxiety measured using the Child Anxiety Life Interference Scale-Preschool Version (CALIS-PV) [43]. The CALIS-PV was adapted from a corresponding questionnaire developed and validated for older children and adolescents [44], with evidence to support its reliability and validity among children aged 3–7 years [43]. Parents rate 18 items on a 5-point scale (0 = not at all to 4 = a great deal; total score range 0–72), including items related to how much being anxious or very shy interferes with their child’s life and their life.
- Child externalising symptoms assessed using the parent-report Strengths and Difficulties-Externalising Scale (SDQ-E; 10 items; total score range 0–20 [45, 46]). The parent-report SDQ is widely used in community and clinical settings and is suitable for 2–17-year-olds.

#### Moderators of primary outcome

The following potential moderators of the absence/presence of an anxiety disorder at 12 months will be assessed at screening/baseline:

- Child age/year group, child gender, child ethnicity, parent gender, parent ethnicity, and family socioeconomic status (using index of multiple deprivation score) measured using a bespoke parent-report socio-demographic questionnaire at screening and baseline.
- Presence/absence of each risk factor (child anxiety symptoms, behavioural inhibition, parent anxiety symptoms) and the number and combination of risk factors on the basis of screening outcome (above/below cut-off) on the PAS, STSC-A, and GAD-7.
- Parental motivation to engage with an online and telephone support programme assessed using a bespoke questionnaire at baseline. Parents will use a 5-point response scale to rate their agreement with 6 statements related to perceived benefits of the programme (e.g. ‘I expect my family would benefit from the online and telephone support programme’), potential concerns (e.g. ‘I am afraid the online and telephone support may take up too much time’), and motivation to use acquired skills (‘I am motivated to use the skills that we would learn from the programme’).

#### Mediators of primary outcome

The following potential mediators of the absence/presence of an anxiety disorder at 12- months will be assessed at 6 weeks and 12 weeks post-randomisation:

- Risk factors (child anxiety symptoms, behavioural inhibition, parent anxiety symptoms) measured using PAS, STSC-A, and GAD-7 total scores
- Additional parent and child behaviours that are intervention targets, including:

(i) Parent overprotection measured using the Parent Overprotection Scale (POS) [47]. The POS consists of 19 items that are designed to assess parenting behaviours that may restrict a child’s exposure to situations that may be perceived as potentially threatening or harmful. Parents rate each item on 5-point (0–4) scale and responses are summed to produce a total score (total score range 0–76) which have demonstrated good psychometric properties among 3–5-year-olds [47] and 7–12-year-olds [48, 49].
(ii) Parenting self-efficacy measured using the 7-item self-efficacy subscale of the Parenting Sense of Competence Scale (PSOC-SE) [50]. Parents rate the extent to which they agree or disagree with 7 statements on a 6-point scale (1 = Strongly Disagree to 6 = Strongly Agree). Items can be summed to provide a total score (range 7–42), with higher scores reflecting higher self-efficacy. The PSOC is a widely used measure of parenting self-efficacy, with evidence to support its reliability [50, 51].
(iii) Child behavioural avoidance measured using the 8-item parent-report version of the Child Avoidance Measure (CAMP) [52]. Items designed to assess a child’s tendency to avoid anxiety-provoking situations are rated on a 4-point scale (0 = never, almost never, or not an issue to 3 = almost always) and summed to provide a total score (range 0–24) which display good psychometric properties among primary-school aged children [52].
(iv) Child intolerance of uncertainty assessed using the 17-item Responses to Uncertainty and Low Environmental Structure (RULES) [53]. Items assess young children’s responses to uncertainty and unstructured situations and for each item parents rate how well the statement describes their child (5-point response scale (1–5); total score range 17–85).
(v) Child coping efficacy measured using an adapted version of the parent-report Coping Questionnaire (CQ-P) [54, 55]. The CQ-P is designed to detect change in coping efficacy during and after CBT interventions and has been used with children as young as 6 years [56]. On the original measure, parents rate their child’s ability to cope in three anxiety-provoking situations identified before starting treatment. In this trial, at baseline we will ask parents to provide three situations where their child feels scared or worried, and then they will rate how well their child is able to cope in each of these situations at baseline and follow-up assessments. Items are rated on a 7-point scale (1-7) and summed to provide a total score (range 3–21).

#### Risks and additional intervention targets

All three risk factors, and parent and child behaviours that are additional intervention targets (parent overprotection, parent self-efficacy, child intolerance of uncertainty, child behavioural avoidance, child coping efficacy) will also be assessed as secondary outcomes, using measures detailed above administered at baseline, 12 weeks, and 12 months post-randomisation.

#### Experiences of screening and the intervention

Interviews will be topic-guided and tailored for each participant group (parents, children, school staff). We will explore participants’ experiences of systematic screening and OSI, including any negative impacts and areas for potential improvement. Audio recordings of interviews will be transcribed verbatim, with any information that could identify an individual removed from transcriptions.

#### Economic outcomes

- Child and parent health-related quality of life (HRQoL) will be assessed using the parent-report version of the Child Health Utility-9D (CHU-9D) [57, 58] and the parent self-reported EQ-5D-5L [59], respectively, at baseline, 12 weeks, and 12 months. The CHU-9D is a preference-based measure of paediatric HRQoL. It includes nine dimensions (worried, sad, pain, tired, annoyed, schoolwork, sleep, daily routine, activities), each with five ordered levels. The EQ-ED-5L [59–61] is a preference-based measure of adult HRQoL. It includes a descriptive system, which covers five dimensions (mobility, self-care, usual activities, pain/discomfort, and anxiety/depression), each with five ordered levels. It also includes a visual analogue scale (VAS), which records the respondent’s self-rated health on a scale ranging from 0 to 100, where the endpoints indicate the worst (0) and the best (100) health the respondent can imagine. The CHU-9D and the descriptive system of the EQ-5D-5L will be used to estimate children and parents’ quality-adjusted life years (QALYs) for use in cost-utility analysis.
- Individual health care and broader resources used during the study period, both by the child and the parent (e.g. health and social care use including mental health service use, time off school (child), time off work (parent)), will be measured using a modified version of the Client Services Receipt Inventory (CSRI) [62], which will be completed by the parent at baseline, 12 weeks, and 12 months. At baseline and 12 week assessments, parents will also be provided with a diary to keep a record of time off school/work and use of services throughout the study duration to facilitate completing subsequent CSRIs.
- To identify and measure resources used in the intervention and screening, we will use “ad hoc” designed therapist, supervisor, and school staff logs.

### Additional measures and information

#### Socio-demographic information

The following school-level demographic information will be collected from the Department for Education website: local education authority area, number of pupils on the roll, percentage of pupils eligible for free schools meals, percentage of pupils on special educational needs support, percentage of pupils with English as an additional language.

A bespoke parent-report questionnaire will be used to collect participant socio-demographic information at screening/baseline (child: month and year of birth, year group, ethnicity, whether their child is fostered or adopted, eligibility for free school meals; parent: age, gender, relationship to child; household: postcode, housing tenure, number of children living in household, parent level of education, parent employment status, parent occupation, income). Parents will also be prompted to provide any changes to household circumstances and employment/occupation at follow-up assessments.

#### Acceptability

Parents will complete a bespoke questionnaire measure at baseline to assess acceptability of screening procedures, and at 12-week and 12-month follow-ups to assess acceptability of trial procedures and (where applicable) OSI. Questions address positive experiences (e.g. ‘Taking part in this study was helpful for me/and or my child’) and negative/adverse experiences (e.g. ‘Taking part in this study was harmful for me and/or my child’).

#### Usual school practice

We will collect information on activities related to social, emotional, and mental health and wellbeing offered in all participating schools at baseline and 12-month follow-up. At baseline, we will collect this information using bespoke questions developed specifically for this study and school staff will be able to opt to answer these questions via an online survey or a telephone/video call with a researcher. We plan to use responses at baseline to further refine questions and procedures for collecting this information at 12 months.

#### Measures used to guide OSI

Parents in the intervention arm will complete the following weekly questionnaire measures as part of the online modules: Child Anxiety Impact Scale (CAIS)/CAIS-global subscale [63], Outcome Rating Scale [64], Goal-Based Outcomes [65], Session Rating Scale [66]. We will also capture data related to OSI usage (e.g. modules completed, online worksheets, and activities completed).

### Planned analysis

#### Primary and secondary clinical outcomes

Baseline and follow-up characteristics and outcome measures will be summarised for each of the intervention and control arms using numbers and percentages for categorical variables and means and standard deviations (or medians and interquartile ranges) for continuous variables. Outcomes at follow-up will be compared between the trial arms using the intention-to-treat principle with participants analysed according to the trial arm the school was randomised to. Marginal models using Generalised Estimating Equations with robust estimates of standard error (specifying an exchangeable correlation structure) will be used to analyse binary outcomes (including the primary outcome) and random effects linear regression models will be fitted to compare continuous outcomes. These methods allow for the correlation between responses from the same cluster. Both unadjusted (crude) estimates of intervention effect and estimates that are adjusted for key prognostic variables will be obtained; the main findings will be based on the adjusted analyses. The main findings will be based on analyses where missing data are imputed using a joint modelling approach based on a multivariate linear mixed effects model that includes random effects for the schools (clusters). Analyses based on using only participants with complete data will also be run to examine sensitivity of the findings to the handling of missing data. Further sensitivity analyses will be undertaken to assess the effect of compliance with the intervention by estimating the Complier Average Causal Effect (CACE) using Instrumental Variable (IV) methods. Tests of interaction using regression-based models will be used to identify factors that moderate the effect of the intervention on the primary outcome, specifying terms for the interaction between trial arm status and the potential moderators. Path analysis models will be fitted to identify factors that mediate the effect of the intervention on the primary outcome. Missing data will be imputed using *R* software and analyses will be carried out using *Stata* and *R* software.

#### Economic outcomes

The economic evaluation will comprise cost-utility (CUA) and cost-effectiveness (CEA) analyses from the NHS perspective (base-case analysis) as per NICE recommendations [67], adopting an intention-to-treat approach. We will adopt a societal perspective in sensitivity analyses in recognition of the fact that the economic costs of mental health have wide consequences beyond the health and social care sectors, including lost education for children, and productivity losses for parents. We will follow best-practice guidelines for conducting our economic evaluation analyses and reporting results [67, 68].^.^ Multiple imputation methods will be adopted to deal with missing data. In the CUA, reported health outcomes will be QALYs gained for the child, as derived from the CHU-9D; and QALYs gained for the parent, as derived from the EQ-5D-5L. In the CEA, the primary clinical outcome will be used, i.e. absence/presence of an anxiety disorder at 12 months post-randomisation established on the basis of the ADIS-P. For each child, components of treatment costs/other health care use and further individual, family, and wider societal costs (as collected using the economic logs completed by wellbeing practitioner/supervisors/ school staff/ parents) will be computed by multiplying units of resource use by their unit costs and then summed to obtain a total cost per patient. Unit costs for health, social care, and other resources will be mainly derived from local and national sources (e.g. PSSRU, 2020 [69]; NASUWT, 2020 [70]) and estimated in line with best practice. Costs will be expressed in pounds sterling at current prices. Given the short time-frame of the trial and follow-up, discounting will not be applied to costs or effects. Statistical methods for combining costs and outcomes will take account of the correlation between costs and outcomes at both the individual level and the cluster level [71, 72]. The economic evaluation outcomes will be expressed as incremental cost per QALY gained in the cost-utility analysis and incremental cost per child free of anxiety diagnoses in the cost-effectiveness analysis. Uncertainty around results will be accounted for and presented using cost-effectiveness acceptability curves [73], as appropriate.

#### Participant experiences

Transcribed interviews will be analysed using thematic analysis [74] to explore experiences of screening and the intervention and capture issues related to acceptability. We anticipate that analysis will be led by one researcher who will meet regularly with co-analysts to discuss candidate themes/subthemes to ensure credible interpretation of the data.

### Data management

Data will be collected, processed, and stored in accordance with the study data management plan, and UK and European data protection laws. We will use REDCap (Research Electronic Data Capture) databases to collect data from participants via online surveys and data entered by researchers. Data not captured online (e.g. diagnostic outcomes) will be entered and checked by two researchers. Each school and participant will be assigned a unique ID to label all study data. A linking document with ID and personal details will be stored on a secure university server, with access restricted to research team members. Audio recordings of diagnostic and qualitative interviews will be stored on the university server until analysis/supervision is complete, and then permanently destroyed.

Pseudononymised trial databases will be cleaned, locked, and signed off prior to sharing with the study statisticians and health economists, and initial data exports will be modified to ensure statistician blinding is maintained where necessary. The University of Oxford’s OneDriveforBusiness will be used to share pseudononymised study data with study statisticians and health economists for analysis. Data files shared with study statisticians for analysis of primary and secondary clinical outcomes will not reveal school/participant arm name.

### Trial governance

The study host and sponsor is The University of Oxford. CC and TR oversee all aspects of the project. MV holds primary responsibility for the economic aspects, OU for the statistical analysis, and CH for the adaptation and implementation of OSI. The Study Management Group (all investigators, including parent representatives FM and BJa) will input into all aspects of the trial through biannual full group meetings and regular subgroup meetings. An independent Trial Steering Committee (TSC) has been convened and will meet at least twice a year to monitor progress and ethical issues, advise the investigators on scientific and management issues, and ensure that there are no major deviations from the study protocol. The TSC includes an independent chair and six independent members (including parent, school, researcher, statistician, health economist, clinician representatives). If the TSC identify concerns that require access to unblinded data, an independent Data Monitoring and Ethics Committee (DMEC) will be convened.

### Dissemination of outcomes

We will disseminate findings from this research as widely as possible to reach academic, clinical, educational, policy maker, and public audiences. All academic publications will be open access and we will share findings through a range of other channels, e.g. newsletters, blogs, study websites, social media, presentations at conferences and events. We will provide schools and participants with a report of study findings, and anonymised study data will be placed in a University repository for reuse by researchers.

## Discussion

This trial aims to evaluate the effectiveness and cost-effectiveness of offering a brief, online parent-led intervention, compared with usual school provision only, for children identified as at risk for anxiety disorders through systematic screening in schools. We also hope to establish who benefits from the intervention and who does not, and how to optimise intervention content going forwards. Increased understanding of how best to prevent the development of anxiety disorders stands to have a substantial impact on children, their families, healthcare providers, and broader society. If effective, and representing good value for money, we hope that this trial will not only advance knowledge about risks for child anxiety disorders and their mechanisms, but will also influence health policy and practice by informing prevention and early intervention practices and provide a model for identification and intervention that can be expanded to other common mental health conditions in children and to other international settings.

It is important to highlight that recruitment for this trial started within the context of the COVID-19 pandemic, and it is possible that disruptions and restrictions related to the pandemic may continue for much of the trial. Data collection and intervention delivery are all conducted online and/or by telephone/video calls and in-person contact with study participants is not required. Nevertheless, it is likely that COVID-19 restrictions will have some impact on recruitment rates, children and parents’ anxiety levels, and the relevance of some assessment questions. We will closely monitor pandemic-related impact and consider appropriate adjustments to procedures as needed to minimise disruption/negative impacts (e.g. starting with a small cohort of schools, adjusting the timing for starting cohorts, amending instructions for questionnaires where appropriate).

## Trial status

School recruitment began in February 2021, and recruitment of participants for screening began in March 2021. We anticipate recruitment will continue through to at least December 2021. This protocol is V2.0 18.6.2021.

## Supplementary Information

**Additional file 1. **SPIRIT Checklist for *Trials*

## Data Availability

Datasets and study materials generated during the current study will be made available in a public repository.

## Abbreviations

ADIS-P: Anxiety Disorder Interview Schedule for Children Parent interview
CACE: Complier Average Causal Effect
CAIS: Child Impact Anxiety Scale
CALIS-PV: Child Anxiety Life Interference Scale-Preschool Version
CAMP: Child Avoidance Measure
CEA: Cost-effectiveness analysis
CHU-9D: Child Health Utility 9 Dimension instrument
CQ-P: Coping Questionnaire
CSR: Clinical Severity Ratings
CSRI: Client Service Receipt Inventory
CUA: Cost-utility analysis
CWP: Children’s Wellbeing Practitioner
DMEC: Data Monitoring and Ethics Committee
DSM-5: Diagnostic and Statistical Manual of Mental Disorder-5
EQ-5D-5L: EuroQol 5 Dimension 5 Level instrument
GAD-7: Generalised Anxiety Disorder Scale-7
IV: Instrumental Variable
NHS: National Health Service
OSI: Online Support and Intervention for child anxiety
PAS: Preschool Anxiety Scale
PI: Principal Investigator
PSO: Parent Overprotection Scale
PSOC-SE: Parenting Sense of Competence Scale
REDCap: Research Electronic Data Capture
RULES: Responses to Uncertainty and Low Environmental Structure
SDQ-E: Strength and Difficulties Questionnaire-Externalising Scale
SPIRIT: Standard Protocol Items: Recommendations for Interventional Trials
STSC-A: Approach subscale of the Short Temperament Scale for Children
TSC: Trial Steering Committee
